# Identification of polycomb repressive complex 1 and 2 core components in hexaploid bread wheat

**DOI:** 10.1186/s12870-020-02384-6

**Published:** 2020-10-14

**Authors:** Beáta Strejčková, Radim Čegan, Ales Pecinka, Zbyněk Milec, Jan Šafář

**Affiliations:** 1 Institute of Experimental Botany, Czech Academy of Sciences, Centre of the Region Haná for Biotechnological and Agricultural Research, Šlechtitelů 31, 77900 Olomouc, Czech Republic; 2 Department of Plant Developmental Genetics, Institute of Biophysics, Academy of Sciences of the Czech Republic, 61200 Brno, Czech Republic

**Keywords:** Polycomb repressive complex, Epigenetics, PRC2, Wheat, Histone methylation

## Abstract

**Background:**

Polycomb repressive complexes 1 and 2 play important roles in epigenetic gene regulation by posttranslationally modifying specific histone residues. Polycomb repressive complex 2 is responsible for the trimethylation of lysine 27 on histone H3; Polycomb repressive complex 1 catalyzes the monoubiquitination of histone H2A at lysine 119. Both complexes have been thoroughly studied in *Arabidopsis,* but the evolution of polycomb group gene families in monocots, particularly those with complex allopolyploid origins, is unknown.

**Results:**

Here, we present the in silico identification of the Polycomb repressive complex 1 and 2 (PRC2, PRC1) subunits in allohexaploid bread wheat, the reconstruction of their evolutionary history and a transcriptional analysis over a series of 33 developmental stages. We identified four main subunits of PRC2 [E(z), Su(z), FIE and MSI] and three main subunits of PRC1 (Pc, Psc and Sce) and determined their chromosomal locations. We found that most of the genes coding for subunit proteins are present as paralogs in bread wheat. Using bread wheat RNA-seq data from different tissues and developmental stages throughout plant ontogenesis revealed variable transcriptional activity for individual paralogs. Phylogenetic analysis showed a high level of protein conservation among temperate cereals.

**Conclusions:**

The identification and chromosomal location of the Polycomb repressive complex 1 and 2 core components in bread wheat may enable a deeper understanding of developmental processes, including vernalization, in commonly grown winter wheat.

## Background

The regulation of gene expression in higher organisms includes a wide range of mechanisms acting at transcriptional, posttranscriptional and posttranslational levels. More complex regulation that is required to coordinate proper gene activity also includes regulation by chromatin remodeling via histone modifications (methylation, acetylation, phosphorylation, and ubiquitination), which lead to specific chromatin changes. Prominent posttranslational changes are histone modifications, which occur on particular amino acid residues. Methylation of lysine 4 on histone H3 (H3K4me) is mainly associated with transcriptional activation, whereas di- and trimethylation of lysines 9 and 27 (H3K9me2 and H3K27me3, respectively) leads to transcriptional repression [1]. H3K9me2, together with small double-stranded RNAs and DNA hypermethylation, contributes to the silencing of repetitive DNA sequences [2, 3]. The repressive epigenetic regulatory processes of genes are usually controlled by Polycomb group proteins (PcG), which are, at the basic level, evolutionarily conserved among plants and animals [4]. Initially identified in *Drosophila melanogaster,* Polycomb repressive complex 1 (PRC1) and 2 (PRC2) are two of the main complexes involved in developmental gene regulation (reviewed in [4–6]). Traditionally, PRC1 and PRC2 have been suggested to work in a hierarchical PRC2 → PRC1 manner [7], but recently, a PRC2-independent function of PRC1 has been suggested [8, 9]. According to the hierarchical model, PRC2 binds to specific DNA sequence motifs called polycomb response elements (PRE) and trimethylates H3 at lysine 27 (H3K27me3) in nearby nucleosomes, recruiting PRC1, which catalyzes monoubiquitination of histone H2A (H2AK119u1) and stabilizes H3K27me3 modification via chromatin remodeling [10]. The PRC2:PRC1-independent model proposes that PRC1 and PRC2 have their own specific adaptor proteins that bind the PRE, and that consequently, PRC1/2 are independently recruited via interactions with their particular adaptor protein [8].

*Drosophila* PRC1 contains four core components, Polycomb (Pc), Polyhomeotic (Ph), Posterior sex combs (Psc) and Sex combs extra (Sce); a fifth component, Sex combs on midleg (Scm), has also been reported (reviewed in [6]). The presence of PRC1 has been unclear in plants until RING-finger proteins were described in *Arabidopsis* [11, 12]*.* In *A. thaliana*, LIKE HETEROCHROMATIN PROTEIN1 (AtLHP1) substitutes for the Pc function [13]. With its chromodomain, LHP1 recognizes and binds histone H3 methylated lysine 27 (H3K27me3) [14]. *A. thaliana B LYMPHOMA Mo-MLV INSERTION REGION 1 HOMOLOG* (*AtBMI1A to C*) are three homologs of Psc, and *REALLY INTERESTING NEW GENE1* (*AtRING1A* and *AtRING1B*) are two homologs of Sce (reviewed in [15]). No Ph homolog has been identified in plants to date [16]. However, plant-specific proteins related to PRC1, such as *A. thaliana* EMBRYONIC FLOWER1 (AtEMF1) [17] or *A. thaliana* VERNALIZATION1 (AtVRN1) [18], have been suggested. EMF1 is involved in the control of shoot architecture and flowering in *Arabidopsis* [19] and interacts with the AtBMI1 and AtRING1 homologs of PRC1 [20, 21]. In contrast, there is no report on the interactions between AtVRN1, which is involved in vernalization in *Arabidopsis* [22], and other PRC1 components to date [23]. Thus, there is no consensus regarding whether VRN1 is a core component of PRC1. Recently, an alternative complex with a PRC1-like function was reported [24]. In *Arabidopsis,* two homologous BAH (Bromo-adjacent homology) domain-containing proteins form a plant-specific complex with EMBRYONIC FLOWER1 (AtEMF1), and this BAH–EMF1 complex reads and effects the H3K27me3 mark and mediates genome-wide transcriptional repression. A homolog of a BAH-domain protein has also been found in monocots (rice), which may indicate its conservation in flowering plants [24]. Genes encoding PRC1 subunits have also been reported in monocots, e.g., *Zea mays* and *Oryza sativa* [23], but not in agronomically important temperate cereals, such as wheat or barley.

The PRC2 complex is formed by four subunits: Enhancer of zeste [E(z)], Extra sex combs (Esc), Suppressor of zeste 12 [Su(z)12] and WD protein p55 [25]; however, similar to PRC1, an additional fifth core component (Jing) has been suggested in *Drosophila* [6]. In plants, PRC2 has been thoroughly studied in *Arabidopsis* (reviewed in [4]). The catalytic activity of PRC2 is histone methylation associated with the SET domain in E(z). Three E(z) homologs have been described to date: CURLY LEAF (CLF) [26], SWINGER (SWN) [27] and MEDEA (MEA) [28]. Similarly, three homologs of Su(z) have been identified: REDUCED VERNALIZATION RESPONSE2 (VRN2) [29], EMBRYONIC FLOWER2 (EMF2) [30] and FERTILIZATION INDEPENDENT SEED2 (FIS2) [31]. The *ESC* homolog *FERTILIZATION INDEPENDENT ENDOSPERM* (*FIE*) is present as a single gene; in contrast, five genes (*MSI1 to MSI5*) have been found for the WD40 p55 homolog (*MULTICOPY SUPPRESSOR OF IRA1*, *MSI*) in *Arabidopsis* [32]. Each of the *Arabidopsis* E(z) and Su(z) homologs functions at different developmental stages (reviewed in [33]). The E(z) homolog MEA is active during early endosperm development [34]; SWN and CLF play a role in vegetative development and vernalization. The initiation of flowering after vernalization is controlled by the flowering repressor *FLOWERING LOCUS C* (*FLC*) [35, 36]. It was also shown that the H3K27me3 level increases and gradually silences *FLC* during vernalization [37]; additionally, *FLC* is completely switched off at the end of the cold period [38]. This status is reset in the next generation, and thus, plants must undergo vernalization to flower.

In *Arabidopsis*, the *clf swn* double mutant completely loses H3K27me3, which indicates the possible inactivation of PRC2 [39]. However, *clf swn* plants form only callus-like structures with occasional somatic embryos [40]. The Su(z) homolog FIS participates in the regulation of the female gametophyte and seed development [41], but the Su(z) homolog EMF2 controls the transition to flowering [42]. Grass PRC2 homologs have been in silico identified in maize, rice and barley [43–49], with functions mainly associated with seed and endosperm development [49, 50]; for a detailed summary, see [51]. Although Kapazoglou et al. [49] identified the barley PRC2 homologs *HvFIE*, *HvE(z)*, *HvSu(z)12a* and *HvSu(z)12b*, *p55* has not been found.

Recently, Lomax et al. [52] identified a *Brachypodium distachyon* mutant without vernalization requirements. A mutation in *Enhancer of zeste-like* (*EZL1*), an ortholog of *A. thaliana CLF*, causes an overall reduction in H3K27me3 and H3K27me2 at *B. distachyon VERNALIZATION1* (*BdVRN1*) and, consequently, earlier flowering without vernalization. A significant reduction in H3K27me3 levels in several regions of *TaVRN1* during vernalization has also been reported in the bread wheat *Triticum aestivum*, correlating positively with the length of the cold period [53]. These findings indicate an important role for PRC2-mediated H3K27me3 deposition in the process of vernalization in grasses.

Despite the socioeconomic importance of bread wheat, our understanding of biological processes has been limited due to the absence of an annotated reference genome until recently, when the International Wheat Genome Sequencing Consortium (IWGSC) published a reference genome of the cultivar Chinese Spring [54]. Overall, the complex wheat genome has proven difficult to decode because of its polyploid nature and high repeat content. Bread wheat (2n = 6x = 42) is a recently formed allohexaploid with a large nuclear genome size (16,974 Mb/1C, [55]) assembled from three homoeologous subgenomes (A, B and D) and with more than 85% of repetitive elements. Thus, deep analyses of genes and their biochemical pathways as well as the molecular basis of central agronomic traits lag behind those of other crops and model plant species, such as *A. thaliana*.

Here, we report the identification and chromosomal location of bread wheat genes encoding the individual subunits of PRC2 and PRC1. We analyzed the mRNA levels of individual genes at different developmental stages and found sequence conservation with other *Triticeae* species, such as *Triticum urartu*, *Aegilops tauschii* and *Triticum dicoccoides*, using a phylogenetic approach. We also discuss the putative role of PRC2 and PRC1 in the vernalization process in bread wheat.

## Results

### In silico identification of wheat PRC2 and PRC1 core components

Using protein sequences of the *Arabidopsis* PcG homologs, we identified wheat components and their respective chromosomal locations. As expected, homoeologs of individual components in all three wheat subgenomes A, B and D were also located. Bread wheat components are designated with the prefix “Ta” representing *Triticum aestivum* followed by A, B or D to indicate the subgenome location. If additional entries were identified on a different chromosome or the same chromosome but at a different position, the respective number was added to distinguish between individual paralogs, for example, TaSu(z)-2A1 (Table 1). The chromosomal positions were validated using the available reference genomes of *T. urartu* (2n = 2x = 14), *T. dicoccoides* (wild emmer wheat, 2n = 4x = 28, accession Zavitan) and *H. vulgare* (2n = 2x = 14, cultivar Morex) (Additional file 1: Table S1).

**Table 1:** Polycomb group core components

*Drosophila*	*Arabidopsis*	Wheat		
PRC2				
*E(z)*	*SWN*	*TaE(z)-4A1 (TraesCS4A02G121300.1)*	*TaE(z)-4B1 (TraesCS4B02G181400.3)*	*TaE(z)-4D1 (TraesCS4D02G184600.3)*
	*CLF*	*TaE(z)-7A1.1 (TraesCS7A02G128300.1)*	*TaE(z)-7B1.1 (TraesCS7B02G028200.2)*	*TaE(z)-7D1.1 (TraesCS7D02G127100.2)*
		*TaE(z)-7A1.2 (TraesCS7A02G128600.1)*	*TaE(z)-7B1.2 (TraesCS7B02G028500.2)*	*TaE(z)-7D1.2 (TraesCS7D02G127400.1)*
	*MEA*	*n/a*		
*Su(z)*	*EMF2*	*TaSu(z)-2A1 (TraesCS2A02G000100.1)*	*TaSu(z)-2B1 (TraesCS2B02G023900.1)*	*TaSu(z)-2D1 (TraesCS2D02G000600.1)*
		*TaSu(z)-2A2 (TraesCS2A02G002500.1)*	*TaSu(z)-2B2 (TraesCS2B02G020400.3)*	*–*
		*TaSu(z)-5A1 (TraesCS5A02G179600.1)*	*TaSu(z)-5B1 (TraesCS5B02G177400.3)*	*TaSu(z)-5D1 (TraesCS5D02G184200.2)*
	*VRN2*	*n/a*		
	*FIS2*	*n/a*		
*ESC*	*FIE*	*TaFIE-7A1 (TraesCS7A02G308300.1)*	*TaFIE-7B1 (TraesCS7B02G377900LC.1*)	*TaFIE-7D1 (TraesCS7D02G084500.1)*
		*TaFIE-7A2.1 (TraesCS7A02G089100.1)*	*–*	*TaFIE-7D2 (TraesCS7D02G305100.1)*
		*TaFIE-7A2.2 (TraesCS7A02G089200.1)*	*–*	*–*
		*TaFIE-4A1 (TraesCS4A02G388400.1)*	*–*	*–*
*p55*	*MSI1*	TaMSI1-A1* (TraesCSU02G072700.1)	*TaMSI1-B1 (TraesCS5B02G378700.1)*	*TaMSI1-D1 (TraesCS5D02G385600.1)*
		*TaMSI1-A2 (TraesCS5A02G331900.1)*	*TaMSI1-B2 (TraesCS5B02G332200.1)*	*TaMSI1-D2 (TraesCS5D02G337800.1)*
PRC1				
*Pc*	*LHP1*	*TaLHP1-A1 (TraesCS7A02G337900)*	*TaLHP1-B1 (TraesCS7B02G249200)*	*TaLHP1-D1 (TraesCS7D02G345200)*
*Psc*	*BMI1A, BMI1B, BMI1C*	*TaBMI1-A1 (TraesCS5A02G378600.1)*	*TaBMI1-B1 (TraesCS5B02G382100.1)*	*TaBMI1-D1 (TraesCS5D02G388500.1)*
		*TaBMI1-A2 (TraesCS5A02G058000)*	*TaBMI1-B2 (TraesCS5B02G065600)*	*TaBMI1-D2 (TraesCS5D02G069800)*
*Sce*	*RING1A*	*TaRING1-A1 (TraesCS3A02G327900.2)*	*TaRING1-B1 (TraesCS3B02G357400.3)*	*TaRING1-D1 (TraesCS3D02G321400.2)*
	*RING1B*	*TaRING2-A1 (TraesCS1A02G315400.1)*	*TaRING2-B1 (TraesCS1B02G327300.1)*	*TaRING2-D1 (TraesCS1D02G315600.1)*
*n/a*	*EMF1*	*TaEMF1-A1 (TraesCS3A02G154500.1)*	*TaEMF1-B1 (TraesCS3B02G180800.1)*	*TaEMF1-D1 (TraesCS3D02G161800.1)*

The table shows genes of PRC2 and PRC1 previously reported in *Drosophila* and *Arabidopsis* and those identified in bread wheat. Each column in wheat contains A, B, and D subgenome homoeologs. EMF1 is a plant-specific PRC1-related component that is not present (n/a) in *Drosophila*. The accession numbers of the respective wheat PcG components are listed in Additional file 1: Table S1. An asterisk (*) indicates that the gene was not assigned to any chromosome based on a BLAST search - the chromosome location was determined by a colinearity with *T. urartu* and *T. turgidum*; a dash (−) indicates that no homolog was identified. The gene ID in brackets corresponds to the IWGSC RefSeq v1.1 gene annotation.

*Enhancer of zeste* [*E(z)*] is located on chromosomes 4 and 7 (Table 1). On chromosome 4, *E(z)* was found on the short arm [*TaE(z)-4A1*] and on the long arm [*TaE(z)-4B1*, *TaE(z)-4D1*]; for chromosome 7, *E(z)* was found on the short arm (Additional file 1: Table S1). The position of *TaE(z)-4A1* on the short arm of chromosome 4A corresponds with the pericentric inversion reported in hexaploid wheat [54, 56]. Two paralogs on the respective short arm on chromosome 7 were identified, separated by only tens of kilobases, suggesting that they originated from a local gene duplication event (Additional file 1: Table S1). Furthermore, as a result of multiple insertions and deletions (indels), paralogs located on chromosome 7A differ by 86 amino acids, and those on chromosomes 7B and 7D differ by 85 amino acids, with the longest indel being 137 amino acids in length (Additional file 2: Fig. S1D).

Kapazoglou et al. [49] reported *Suppressor of zeste* [Su(z)] homologs in barley, located on chromosomes 2H and 5H. Similarly, we found wheat homologs on chromosomes 2 and 5. Interestingly, two homologs were identified on chromosomes 2AS and 2BS but only one on 2DS (Table 1). All three homoeologs of group 5 are located on the long arm. The bread wheat diploid progenitor *T. urartu* has only the A genome, and we identified two homologs on the short arm of chromosome 2 at positions ≈ 1.5 Mb and ≈ 2.4 Mb and another on the long arm of chromosome 5. Wild emmer wheat accession Zavitan also carries two homologs on 2AS and one on 2BS together with homologs on 5AL and 5BL (Additional file 1: Table S1).

Two proteins encoded by the genes *TaSu(z)-2A2* and *TaSu(z)-2B2* carry an insertion of 32 amino acids. This insertion was also found in proteins encoded by the *TRIDC2AG000370.14* gene in *T. dicoccoides* and by the *H. vulgare* gene *HORVU.MOREX.r2.2HG0078790.1* located on chromosome 2 (Additional file 2: Fig. S1G).

The Esc subunit reported in *Drosophila* has been designated *FERTILIZATION INDEPENDENT ENDOSPERM1* (*HvFIE1*) in barley [49], and we followed this style and named the wheat homologs *TaFIE*. We found two homologs on 7AS (*TaFIE-7A2.1* and *TaFIE-7A2.2*) and one on 7AL (*TaFIE-7A1*) (Table 1 and Additional file 1: Table S1). Chromosome 7D harbors one gene located on the short arm (*TaFIE-7D1*) and one gene on the long arm (*TaFIE-7D2*). Initially, no 7B homolog was localized using the reference sequence of Chinese Spring by IWGSC. Surprisingly, a paralog was found in the distal part of the long arm of chromosome 4. This corresponds with the fact that this region of chromosome 4 contains a portion of chromosome 7B [56]. Reciprocal BLAST with the 4AL homolog (TaFIE-4A1) showed high similarity with genes previously located on 7AL/7BL in Zavitan and with the barley gene on the 7H chromosome. The predicted barley protein was annotated as FIE [57, 58]. Later, we identified the 7BL homolog TRIAE_CS42_7BL_TGACv1_580129_AA1912160.1 using a BLAST search in the Ensembl plant database using data from wheat genome assembly by TGAC [59] (Additional file 1: Table S1).

The p55 subunit, which contains WD40 domains (same as FIE) together with the N-terminal domain of the histone-binding protein RBBP4, has been designated MSI1 (MULTICOPY SUPPRESSOR OF IRA1) in *Arabidopsis*. In bread wheat, two orthologs (*TaMSI1*) are present on each chromosome of group 5, with one exception: one of the best BLAST results was not anchored to any chromosome (*TraesCSU02G072700*). Comparison with the sequences of *T. urartu* and *T. turgidum* revealed high identity with the 5AL chromosome; therefore, we designated this unassigned accession *TaMSI1-A1,* suggesting its location on chromosome 5A (Table 1 and Additional file 1: Table S1).

However, the localization of wheat PRC1 components was more complicated, as they have not been described in cereals thus far, rendering validation of the results difficult. Therefore, we used the reference sequence of *H. vulgare* containing annotations of predicted proteins.

*LIKE HETEROCHROMATIN PROTEIN1* (*LHP1*) wheat homoeologs were found on the long arm of chromosome 7 and *BMI1* homologs on both short and long arms of chromosome 5. *Arabidopsis* has three *BMI1* homologs (*AtBMI1A* to *AtBMI1C*), but BLAST of *AtBMI1A* and *AtBMI1B* identified the same genes in wheat located on the long arm of chromosome 5. Surprisingly, a BLAST search of *AtBMI1C* identified not only the same wheat homologs but also other paralogous genes located on the short arm. The genes on the short arm correspond to the position of the barley gene, also on the short arm of chromosome 5H. This gene was annotated as *Ubiquitin ligase DREB2A-INTERACTING PROTEIN2* (*DRIP2*, a synonym for *BMI1*) [58] and corresponds to the *Arabidopsis* designation. The genes on the long arm correspond with the position of the barley gene also annotated as *Ubiquitin ligase DRIP2* [58] and located on the long arm of chromosome 5H.

*RING1* homologs were found on the long arm of all three chromosomes of group 3. *RING2* is present on the long arm of all three chromosomes of group 1.

The wheat homolog TaEMF1 was not identified when the *Arabidopsis* protein sequence was used in a BLAST search. However, homologous proteins with genes located on chromosomes 3A, 3B and 3D were found when the EMF1 protein sequence of *Z. mays* was used [23]. The positions of these genes correlate with the location of *HvEMF1* in barley, suggesting that they may be homologs of *AtEMF1*.

**Fig. 1:**
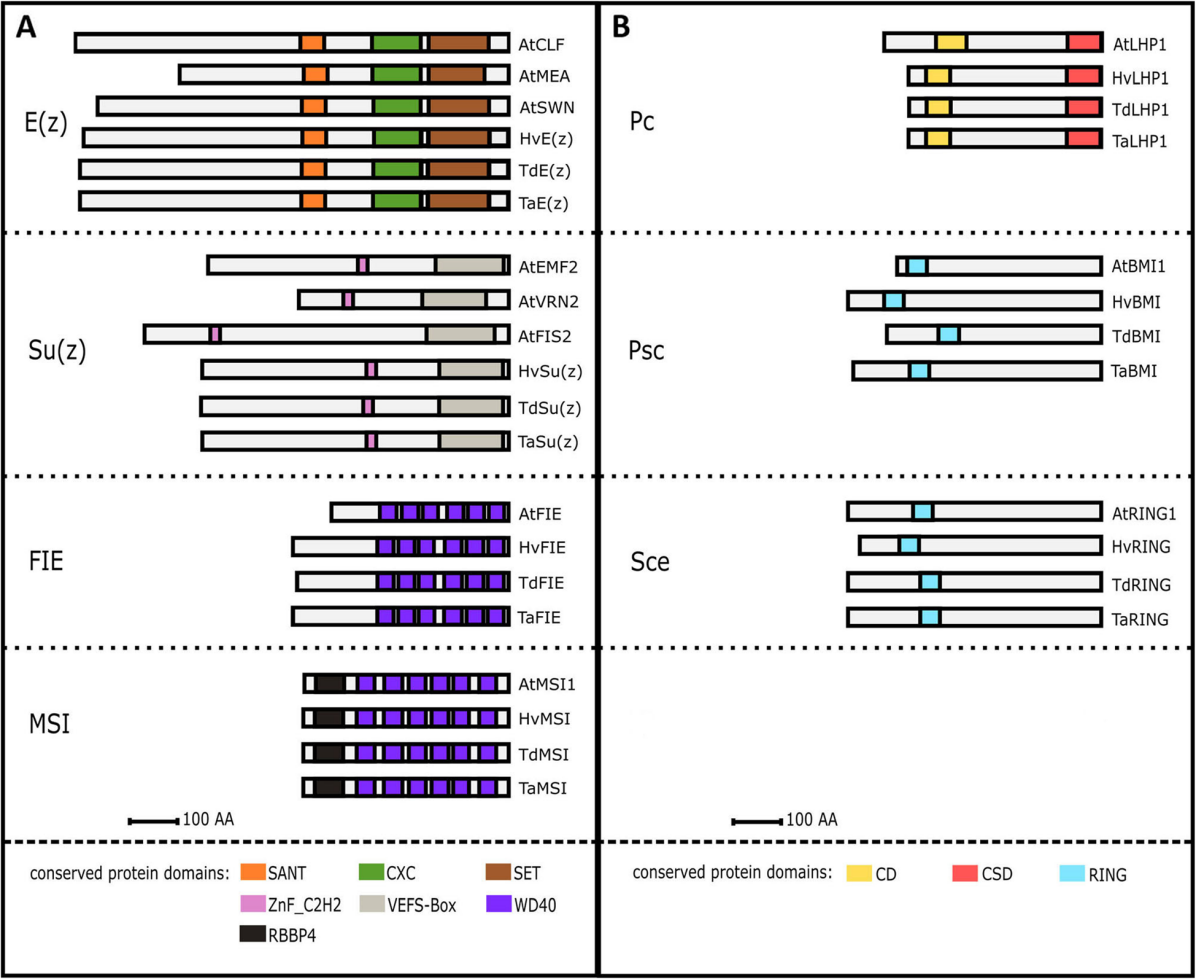
Schematic representation of the conserved protein domain architecture of Polycomb group (PcG) complexes. The in silico identification of the PRC2 and PRC1 core components in hexaploid wheat was supported by protein alignment with known homologs from *Arabidopsis* and barley PRC2 and PRC1 and by prediction of main functional protein domains. Homologs of the PRC2 (**a**) and PRC1 (**b**) core subunits share highly conserved protein domains among *Arabidopsis thaliana* (At), *Hordeum vulgare* (Hv), *Triticum dicoccoides* (Td) and *Triticum aestivum* (Ta). Proteins in the figure are representatives of each homologous group from Hv, Td and Ta, which share the same domains and differ only by protein length

We also identified the main protein domains for individual PcG wheat components (Fig. 1). Comparison of bread wheat with *Arabidopsis*, *H. vulgare* and *T. dicoccoides* showed high domain conservation, which further supported the accuracy of the wheat homolog identification.

### Phylogenetic analysis

**Fig. 2:**
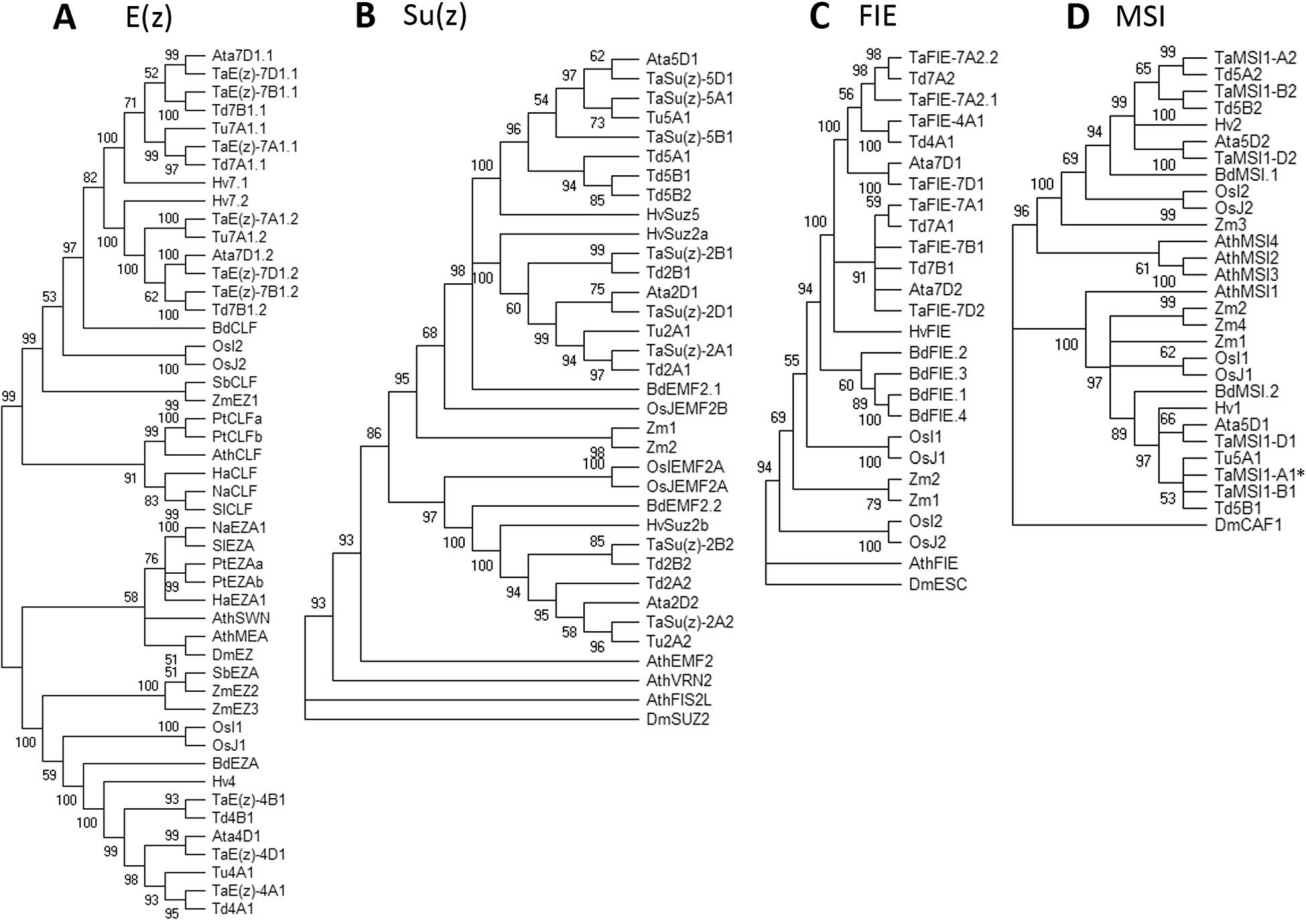
Phylogenetic analysis of the plant PRC2 components E(z) (**a**), Su(z) (**b**), FIE (**c**) and MSI (**d**). The analysis was performed using the maximum likelihood method and JTT matrix-based model in MEGA X. The bootstrap consensus tree was inferred from 1000 replicates. E(z) tree is midpoint rooted. Su(z), FIE and MSI trees are rooted in the outgroup *Drosophila melanogaster* (Dm). *Aegilops tauschii* (Ata), *Arabidopsis thaliana* (Ath), *Brachypodium distachyon* (Bd), *Helianthus annuus* (Ha), *Nicotiana attenuata* (Na), *Populus trichocarpa* (Pt), *Solanum lycopersicum* (Sl), *Sorghum bicolor* (Sb), *Hordeum vulgare* (Hv), *Oryza sativa indica* (OsI), *Oryza sativa japonica* (OsJ), *Triticum aestivum* (Ta), *Triticum dicoccoides* (Td), *Triticum urartu* (Tu) and *Zea mays* (Zm). An asterisk (*) indicates the gene not assigned to any chromosome based on a BLAST search - the chromosome location was determined by a colinearity with *T. urartu* and *T. turgidum*

**Fig. 3:**
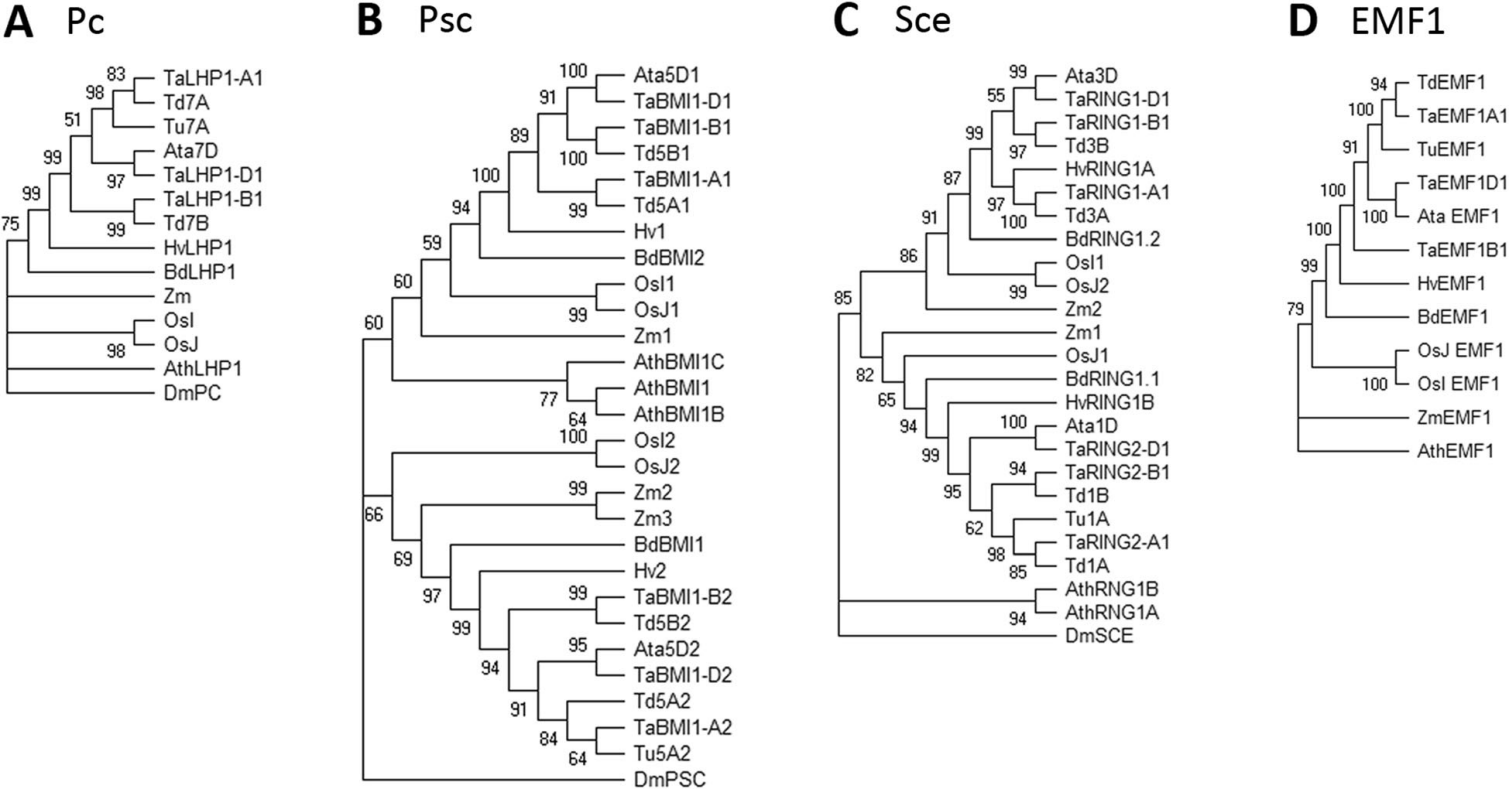
Phylogenetic analysis of the plant PRC1 components LHP1 (**a**), RING1 (**b**), BMI1 (**c**) and EMF1 (**d**). The analysis was performed using the maximum likelihood method and JTT matrix-based model in MEGA X. The bootstrap consensus tree was inferred from 1000 replicates. Trees are rooted in the outgroup *Drosophila melanogaster* (Dm), with the exception of the EMF1 tree, which is rooted in *Arabidopsis thaliana* (Ath). *Aegilops tauschii* (Ata), *Brachypodium distachyon* (Bd), *Hordeum vulgare* (Hv), *Oryza sativa indica* (OsI), *Oryza sativa japonica* (OsJ), *Triticum aestivum* (Ta), *Triticum dicoccoides* (Td), *Triticum urartu* (Tu) and *Zea mays* (Zm)

Phylogenetic trees of both PRC2 and PRC1 wheat components were constructed to reveal the evolutionary relationships among *Arabidopsis*, barley, rice, maize, all bread wheat homologs and bread wheat progenitors (Figs. 2 and 3).

Phylogenetic analysis showed that wheat E(z) homologs, located on chromosomes 4 and 7, fell into separate clades, one including AtSWN and the other including AtCLF, respectively. This suggests that *E(z)* genes on wheat chromosome 4 are putative orthologs of *AtSWN* but that genes on chromosome 7 are putative orthologs of *AtCLF* (Fig. 2a).

*Su(z)* genes were found on chromosomes 2 and 5. The genes on chromosome 2 clustered in one clade, and genes on chromosome 5 clustered into the second clade. The phylogenetic analysis suggests that all *Su(z)* are orthologous to *AtEMF2* (Fig. 2b).

Homologs of *FIE* are located on chromosome 7, but the best BLAST hit was for chromosome 4A. Interestingly, the homolog on the 4AL chromosome (*TaFIE-4A1*) fell into the same clade with the 7AS chromosome homologs (*TaFIE-7A2.1* and *TaFIE-7A2.2*) and not in the clade with the 7AL homolog (Fig. 2c).

*MSI* homologs were found to be in two positions on the long arm of chromosome 5, except for *TraesCSU02G072700*, which was not assigned to any chromosome (Additional file 1: Table S1). However, phylogenetic clustering of this unanchored gene in the same clade together with *TaMSI1-B1* and *TaMSI1-D1* suggests that it may represent the *TaMSI* copy on the 5A chromosome (Table 1).

The phylogenetic analysis of PRC1 components was unremarkable: wheat LHP1 homologs clustered according to subgenomes A, B and D. Although *Arabidopsis* has three BMI1 homologs, wheat BMI1 homologs were grouped into only two clades. This was in agreement with our findings based on alignment (Additional file 1: Table S1). RING homologs clustered into two clades according to their location on chromosomes 1 and 3 (Fig. 3b).

### RNA-seq analysis suggests conserved transcriptional patterns of A, B and D homoeologs

**Fig. 4:**
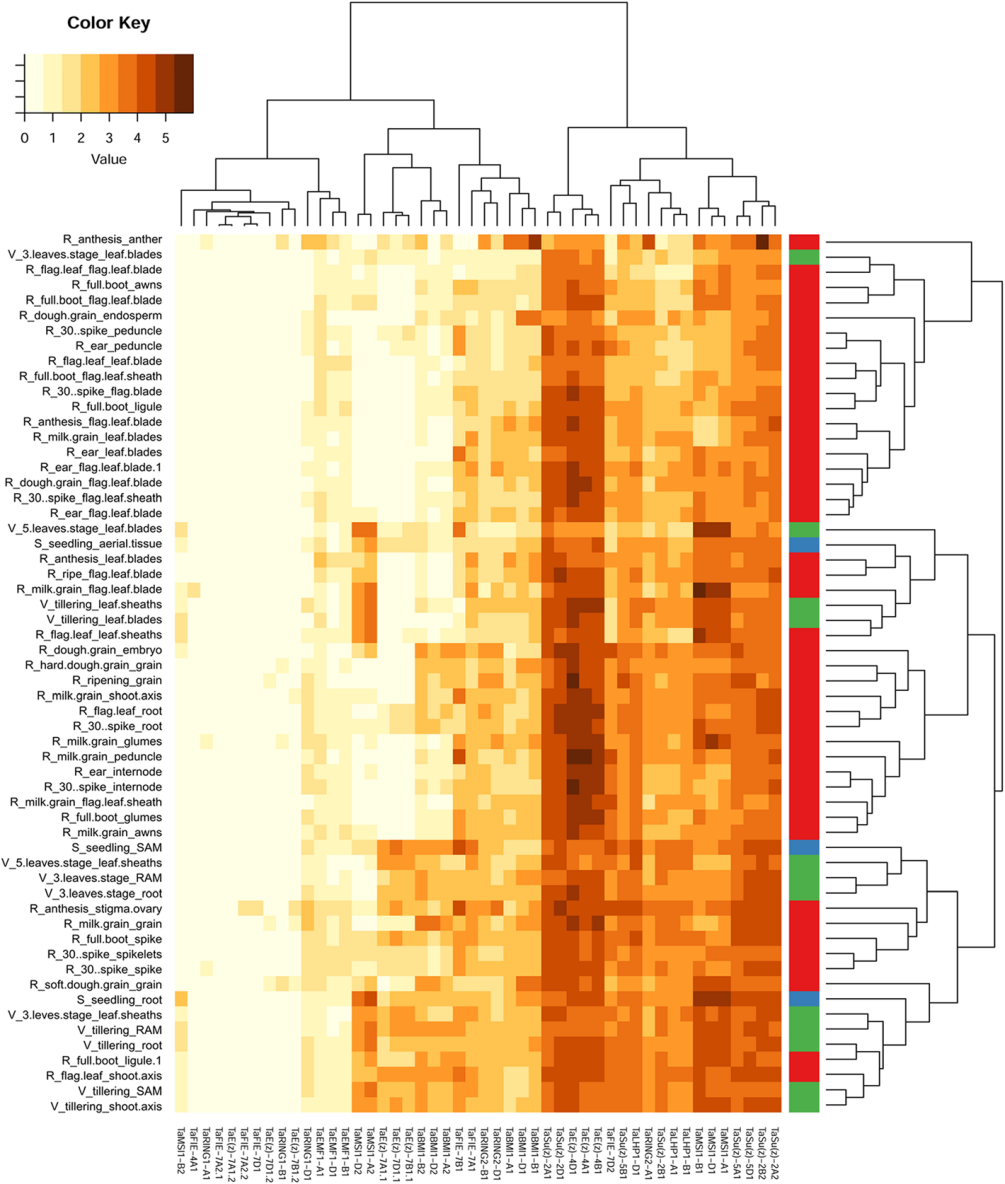
Heat map of PRC1 and PRC2 mRNA levels at different wheat developmental stages. The publicly available RNA-seq data of candidate genes from the cultivar Azhurnaya were clustered based on the transcription profile similarities between the genes (rows) and tissues (columns). Each tissue is characterized as “high-level age_age_tissue”. The high-level stages S – seedling (blue), V – vegetative (green) and R – reproductive (red) are also highlighted by a horizontal color stripe. For a detailed description of the developmental samples and input values, see Additional file 3: Table S2. The color key shows transcripts per million (TPM) after log2 transformation

To estimate transcriptional activity and potential tissue specificity of individual PRC1 and PRC2 subunits, we performed transcriptomic analysis using publicly available RNA-sequencing data for 58 bread wheat developmental stages and tissues from the Azhurnaya accession (expVIP database). Transcripts per million (TPM) values were extracted for all of the above-described genes, clustered based on the similarity of their transcriptional profiles over the tissues and visualized in heat maps (Fig. 4 and Additional file 3: Table S2). TPM values were used after log2 transformation, which allows for easier analysis of many genes with low transcription levels.

We found that the homoeologs within the A, B and D subgenomes frequently showed highly similar transcriptional profiles (e.g., *TaE(z)-4A1, B1, D1*; *TaE(z)-7A1.2, B1.2, D1.2*; *TaBMI1-A1, B1, D1*; and *TaBMI1-A2, B2, D2*; *TaMSI1-A1, B1, D1*). This suggests that the developmental regulation established in the progenitor species still exists in the subgenomes of modern wheat and indicates a low degree of functional differentiation between homoeologous gene copies. A possible exception is that *Su(z)-2B2*, for which 61.82 TPM in anthers (R_anthesis_anther) was obtained, had by far the highest value among all genes in the analysis. Indeed, this mRNA level was 5-fold higher than for its homoeolog *Su(z)-2A2* (TPM 12.39) at the same experimental point. However, both genes showed similar mRNA levels in all other tissues (note that *Su(z)-2D2* was not found in the *T. aestivum* genome). Although the RNA-seq data provided a solid support for the transcription of many PRC1 and PRC2 genes, there were also copies that were hardly transcribed in the set of the analyzed tissues, and this held true even for the entire homoeologous group. For example, *TaE(z)-7A1.2, B1.2,* and *D1.2* copies, representing orthologs of *Arabidopsis CLF*, were largely not expressed throughout development; in contrast, the *TaE(z)* homoeologs on chromosome 4, representing orthologs of *Arabidopsis SWN,* were among the genes with the highest TPM values. A slightly different pattern was observed for *TaMSI1-A2, B2* and *D2* and *TaMSI1-A1, B1* and *D1*, representing tissue-specific and general MSI groups, respectively. However, such correlations were not universally applicable to all homologs of one PRC1 or PRC2 subunit. Clustering by tissues (log2 plot) revealed three main groups, though the differences were relatively few. The first two blocks (from left to right in Fig. 4) consisted mainly of tissues from plants in the reproductive stage and were characterized by the expression of only specific copies. Conversely, the third cluster contained more tissues from seedling and vegetative-stage plants, which expressed the highest number of PRC1 and PRC2 components.

## Discussion

Plant PcG proteins participate in developmental processes, for example, the transition from the vegetative to the generative stage, flowering and seed development [31, 60, 61]. PcG proteins form groups of Polycomb repressive complexes such as PRC1 and PRC2**.** PRC2 controls chromatin remodeling through the methylation of histone H3K27 [5]. This epigenetic marker of repressed genes is quite common. It has been reported that nearly 4500 (16%) genes in *Arabidopsis* carry the repressive mark H3K27me3 [62, 63]. In monocots, many genes are also marked with H3K27me3. Interestingly, a significant level of concurrence between the repressive mark H3K27me3 and transcription level has been reported in rice, where the majority of H3K27me3 marks (almost 85%) is associated with genic regions. In fact, nearly 53% of H3K27me3-marked genes are expressed, and it was revealed that the gene expression level correlated with the ratio of H3K4me3/H3K27me3 and H3K27me3/H3K4me3 [64]. In maize, H3K27me3 is also present mostly in gene-dense chromosome arms and it targets genes with an important regulatory role [65]. In barley, high densities of H3K27me3 were found in telomere-proximal regions, covering both genes and intergenic DNA, where this mark specifies facultative heterochromatin. Similar to rice and maize, H3K27me3 preferentially covers unexpressed genes but is not exclusive to them and can also be found on some transcriptionally active genes [66]. Despite the possibility of such a complex pattern, potential artifacts caused by tissue-specific differences in H3K27me3 and/or different sensitivities of the ChIP and transcriptomic methods may occur.

Conservation of H3K27me3 targets among plant species has been suggested. The targets of H3K27me3 in maize [65] were compared with genes marked with H3K27me3 in *Arabidopsis* [39] and rice [64]. It was found that 34% of maize genes that have homologs in *Arabidopsis* were marked with H3K27me3 in both plants. The number of homologous genes marked with H3K27me3 in both monocot species (rice and maize) was almost two times higher than that in *Arabidopsis* [65]. PRC2 also plays a key role in the vernalization response in *Arabidopsis*. Before vernalization, expression of the major flowering promoter *FLOWERING LOCUS T* (*FT*) is repressed by high levels of *FLC*, but cold treatment triggers PRC2-dependent silencing of *FLC*, which is associated with increased levels of H3K27me3 [37, 67]. When *FLC* becomes inactive, expression of *FT* is initiated and triggers the transition to flowering (reviewed in [68]). In contrast, H3K27me3 marks are present at high levels before vernalization in temperate cereals [52, 53, 69], possibly due to PRC2 activity, as suggested by [70]. This may result in chromatin compaction and *VRN1* repression. During the cold period, the H3K27me3 mark disappears, resulting in chromatin remodeling, which may enable expression of *VRN1*. Consequently, the transition from the vegetative to the reproductive stage can occur. The study of molecular mechanisms such as vernalization is hampered by a lack of detailed information about PcG components in bread wheat. Based on homology searches, we identified and located putative PRC2 and PRC1 genes in bread wheat. Most of the subunits were found to be homoeologs in all three wheat subgenomes (A, B and D).

The chromosomal positions of the wheat PRC2 components corresponded with the previously reported PRC2 genes in barley [49]. Interestingly, several paralogs were found on the same chromosome, and paralogs located on different chromosomes were also found. These multiple sites could be explained by the allohexaploid nature of the wheat genome, which has undergone frequent chromosomal rearrangements. Comparison between individual paralogs also revealed shortened proteins (Additional file 4: Table S3, Additional file 2: Fig. S1) and distinct low to high expression levels. These findings indicate the possible alteration and/or subfunctionalization of the genes. We also identified paralogs that differ with regard to the distance between individual copies. *TaSu(z)-2A1* and *TaSu(z)-2A2* are separated by more than 1.1 Mb, whereas two copies of *TaFIE* genes (*TaFIE-7A2.1* and *TaFIE-7A2.2*) are separated only by a region of 37 kb (Additional file 1: Table S1), which indicates that different mechanisms contribute to gene duplications in wheat. Unfortunately, their expression level based on the expVIP database is minimal.

Interestingly, E(z) paralogs were identified on chromosome groups 4 and 7. A translocation between chromosomes 4 and 7 has been reported [54, 56]. Briefly, the structure of present-day wheat chromosome 4 is an illustrative example of dynamic chromosomal rearrangements within the allohexaploid wheat genome. The final composition of the chromosome resulted from the pericentric inversion of the ancient long arm, which became a modern short arm, and the subsequent translocation from 5AL and 7BS completed the rearrangement of the chromosome. In agreement with this, the copy of the *TaFIE-4A1* gene maintained a closer phylogenetic relationship to the homologs on the 7AS chromosome (*TaFIE-7A2.1* and *TaFIE-7A2.2*) (Fig. 2c).

Moreover, the phylogenetic analysis revealed that genes on chromosome 4 are putative orthologs of *AtSWN* but that genes on chromosome 7 are closer to *AtCLF*. Protein alignment of conserved domains from *Arabidopsis* SWN and CLF with domains from TaE(z) revealed nine independent diagnostic changes of amino acids in the catalytic SET domain. These nine positions are shared by AtSWN and TaE(z) copies on chromosome 4 versus AtCLF and TaE(z) copies on chromosome 7 (Additional file 5: Fig. S2). This indirectly suggests that CLF- and SWN-like proteins already existed prior to the evolutionary split of monocots and dicots [71]. CLF and SWN are largely functionally redundant in *Arabidopsis,* and their simultaneous knockout in plants results in the production of callus-like structures containing somatic embryos [72]. Currently, the extent of functional redundancy between the *TaSWN-like* and *TaCLF-like* groups is unknown, but *TaSWN-like* homoeologs are more strongly expressed than are *TaCLF-like* homoeologs, which contrasts with the pattern in *Arabidopsis* [73]. There was also a substantial difference in mRNA levels (up to 11-fold) between *CLF-like* paralogs on chromosome 7, which may indicate that the *cis*-regulatory elements of some copies were either mutated or lost. Future experiments will reveal whether such copies may be either subfunctionalized at the tissue-specific level or progressing toward removal from the bread wheat genome. Analysis of the expression profile showed that not all paralogs representing individual core components were expressed similarly, though there was always at least one gene with a high expression level. This may be because the paralog sequences were not identical (Additional file 2: Fig. S1); therefore, their function and expression might be altered.

Unlike the identification of LHP1, RING1 and BMI1, which assemble the core components of plant PRC1, the identification of other plant-specific proteins that may be part of this complex was difficult. The chemical properties and functions of EMF1 are similar to those of Psc in *Drosophila* and its ortholog, BMI1, in *Arabidopsis* [74]. The poorly conserved sequence of EMF1 does not display significant homology with any other proteins of known function [19]. There are no annotated domains in EMF1, but five conserved motifs shared by the entire EMF1 orthologous group were predicted [17, 23]. Despite the presence of EMF1 in both monocots and eudicots [17, 19, 23], no direct homolog was found in *T. aestivum* using the EMF1 protein sequence from *Arabidopsis* for homology searches. Therefore, we used a sequence of a monocot plant (maize), suggesting that EMF1 is less conserved among dicots and monocots. *AtVRN1*, which was assigned in previous studies to PRC1 [18, 75], was shown to be absent in monocots [23]. In *Arabidopsis*, *AtVRN1* plays an important role in vernalization. It should be emphasized that the *VERNALIZATION1* (*VRN1*) gene in wheat is not related to *VRN1* in *Arabidopsis* but is homologous to *APETALA1, CAULIFLOWER* and *FRUITFUL* (*AP1, CAL,* and *FUL*), with no role in *Arabidopsis* vernalization [76]. However, when the AtVRN1 protein sequence from *Arabidopsis* was used for a homology search in wheat, similar proteins with genes located on chromosomes 5A, 5B and 5D were obtained. These proteins contain four B3 domains, whereas the AtVRN1 protein in *Arabidopsis* contains only two domains. In summary, all core subunits of PRC1 (consisting of LHP1, RING1, and BMI1 in monocots) in bread wheat were identified. The identification of the plant-specific proteins EMF1 and VRN1 remains less evident. Individual subunits of PRC1 also share conserved protein domains between paralogs, but not all paralogs had the same expression level, indicating differentiation at the cis-regulatory level.

## Conclusions

The identification of individual PcG components in bread wheat will help to reveal the molecular mechanisms of important biological processes. More detailed studies (expression studies, sequence variation among wheat cultivars, etc.) will be necessary to reveal the possible functional divergence of single genes, including paralogs, and their putative role in the formation of Polycomb repressive complexes affecting plant development.

## Methods

### In silico PcG component identification

*T. aestivum* PcG component protein sequences were obtained by BLAST searches of the *T. aestivum* genome in Ensembl Plants (http://plants.ensembl.org/index.html) using *A. thaliana* protein sequences with default parameters. Protein sequences for all studied species that were not available in databases were in silico reconstructed from the genomic sequences according to the *T. aestivum* reference (cultivar Chinese Spring) obtained from Ensembl Plants by local blastn with genomic data of *T. urartu* and *Ae. tauschii*. Data for *T. dicoccoides* were obtained from Ensembl Plants. The obtained nucleotide sequences were aligned to the *T. aestivum* sequence by MAFFT multiple aligner (version 1.3.3) in Geneious 8.1.9 software https://www.geneious.com using default settings. After alignment of genomic sequences, coding sequence (CDS) regions were extracted and translated into proteins. Some genomic sequences are not well assembled, and thus, a sequence corresponding to the reference was sometimes scattered to several scaffolds/contigs. Such genes were reconstructed by extracting partial sequences from several scaffolds, concatenating the CDS regions and translating them into proteins (Additional file 4: Table S3).

Protein sequences for *Hordeum vulgare* were obtained from GenBank https://www.ncbi.nlm.nih.gov/ and barley DB [58]; proteins for *B. distachyon*, *Helianthus annuus*, *Nicotiana attenuata*, *Oryza sativa japonica*, *Oryza sativa indica, Populus trichocarpa*, *Solanum lycopersicum* and *Z. mays* were obtained from UniProt (https://www.uniprot.org/) and Ensembl Plants. All sequences used in the phylogenetic studies are provided in Additional file 4: Table S3.

Reciprocal BLAST searches of identified wheat PcG proteins were performed against the *A. thaliana* database TAIR10 within EnsemblPlants (https://plants.ensembl.org/Arabidopsis_thaliana/Info/Index) to validate the results.

### Phylogenetic analysis

Protein alignments for phylogenetic analysis were conducted in MEGA X [77] by ClustalW. For all genes in the PRC1 and PRC2 complexes, the evolutionary history was inferred using the maximum likelihood method and JTT matrix-based model [78] in MEGA X [77]. The bootstrap consensus tree inferred from 1000 replicates [79] is taken to represent the evolutionary history of the taxa analyzed [79]. Sequences of *Drosophila* PcG proteins were used as outgroups for all trees besides EMF1 where *Arabidopsis* sequence was used as outgroup. All phylogenetic trees were rooted in the outgroup except E(z), which were rooted at the midpoint.

### Transcriptomic analysis

The RNA-seq database “expVIP” http://www.wheat-expression.com was used as a data source for expression analysis of individual PcG core subunits [80, 81]. We used data collected from roots, leaves/shoots, spikes and grains of the spring wheat cultivar Azhurnaya at 58 different time points, corresponding to a total of 22 tissues or organs (Additional file 3: Table S2). The data for the Azhurnaya cultivar represent the developmental time-course, and only data collected from three and more biological replicates were used. Heatmaps were constructed in R software (https://www.r-project.org/) using gplots, heatmap3 and RColorBrewer packages. Both the genes and the developmental stages were clustered based on the similarity of their mRNA amounts at different experimental points.

### Protein domain identification

The SMART http://smart.embl.de/ (in mode normal SMART) [82] and PFAM http://pfam.xfam.org/ [83] protein databases were used to predict conserved protein domains of the PRC2 and PRC1 components of *A. thaliana*, *H. vulgare*, *T. dicoccoides* and *T. aestivum*. A multiple sequence alignment of all found homologous proteins for each PRC2 and PRC1 subunit of *A. thaliana*, *H. vulgare*, *T. dicoccoides* and *T. aestivum* was carried out using MAFFT *v7.388* [84, 85].

## Electronic supplementary material

**Additional file 1 **: Table S1. List of individual PcG components identified in *Triticum aestivum* and their homologs in *Triticum urartu*, *Triticum dicoccoides* and *Hordeum vulgare*. The table shows that PcG components are conserved among cereals. (XLSX 21 kb)

**Additional file 2 **: Fig. S1. This figure shows the protein alignments of plant PRC1 and PRC2 core components. The alignment contains protein sequences of *Triticum aestivum*, *Triticum dicoccoides*, *Hordeum vulgare,* and *Arabidopsis thaliana.* Conserved protein domains are highlighted in different colors. (PDF 13 Mb)

**Additional file 3 **: Table S2**.** RNA-seq data of bread wheat PcG genes used for transcriptomic analysis. All data were collected from wheat variety Azhurnaya and sorted according to the main stage (seedling vegetative stage, reproductive stage) with further refining to individual developmental stages as well as sorted by the main tissue (roots, leaves/shoots, spikes, and grains). (XLSX 51 kb)

**Additional file 4 **: Table S3**.** Protein sequences of PcG components used for phylogenetic analysis. The table contains both monocot and dicot species and has been identified using BLAST. (SLSX 160 kb)

**Additional file 5 **: Fig. S2**.** This figure shows protein alignment of E(z) homologs showing nine amino acid exchanges in the SET domain, allowing the division of TaE(z) paralogs into SWN-like and CLF-like groups. (PDF 661 kb)

## Data Availability

All data generated or analyzed during this study are included in this published article [and its supplementary information files]. All data were obtained from publicly available databases (NCBI https://www.ncbi.nlm.nih.gov/, EnsemblPlants http://plants.ensembl.org/index.html and expVIP http://www.wheat-expression.com/).

## Abbreviations

AP1: APETALA1
BAH: Bromo-adjacent homology
BMI1: B LYMPHOMA Mo-MLV INSERTION REGION 1 HOMOLOG
CAL: CAULIFLOWER
CLF: CURLY LEAF
DRIP2: DREB2A-INTERACTING PROTEIN2
E(z): Enhancer of zeste
EMF: EMBRYONIC FLOWER
Esc: Extra sex combs
EZL1: Enhancer of zeste-like
FIE: FERTILIZATION INDEPENDENT ENDOSPERM
FIS2: FERTILIZATION INDEPENDENT SEED2
FLC: FLOWERING LOCUS C
FT: FLOWERING LOCUS T
FUL: FRUITFUL
LHP1: LIKE HETEROCHROMATIN PROTEIN1
MEA: MEDEA
MSI: MULTICOPY SUPPRESSOR OF IRA
Pc: Polycomb
PcG: Polycomb group proteins
Ph: Polyhomeotic
PRC: Polycomb repressive complex
PRE: Polycomb response elements
Psc: Posterior sex combs
RBBP4: Retinoblastoma-binding protein 4
RING1: REALLY INTERESTING NEW GENE1
Sce: Sex combs extra
Scm: Sex combs on midleg
Su(z): Suppressor of zeste
SWN: SWINGER
TPM: Transcripts per million
VRN1: VERNALIZATION1
VRN2: REDUCED VERNALIZATION RESPONSE2
