# Supplementary material for: Identification of polycomb repressive complex 1 and 2 core components in hexaploid bread wheat
Source: BMC Plant Biol. 2020 Oct 14;20(Suppl 1):175. doi: 10.1186/s12870-020-02384-6 (PMC7557041; doi:10.1186/s12870-020-02384-6)
Supplement: Supplementary file 5 — Additional file 5 : Fig. S2. This figure shows protein alignment of E(z) homologs showing nine amino acid exchanges in the SET domain, allowing the division of TaE(z) paralogs into SWN-like and CLF-like groups. (PDF 661 kb) [file 12870_2020_2384_MOESM5_ESM.pdf]

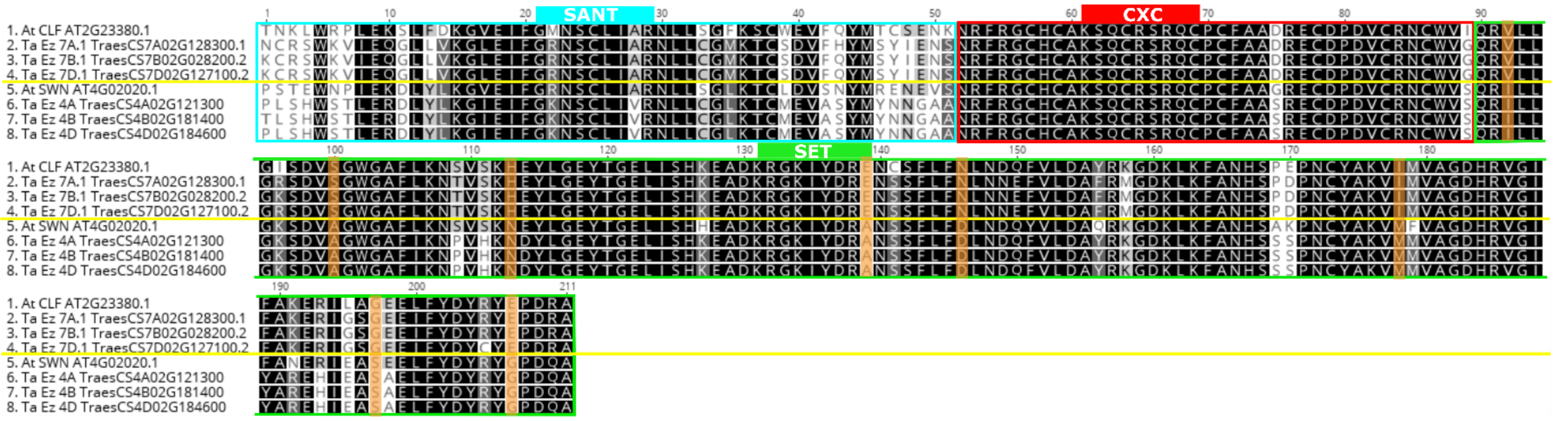

Figure S2. Nine aminoacid exchanges in SET domain corresponding with division of TaE(z) paralogs into SWN- and CLF-like groups are highlighted with orange color.
